# Label-free affinity screening, design and synthesis of inhibitors targeting the *Mycobacterium tuberculosis* L-alanine dehydrogenase

**DOI:** 10.1371/journal.pone.0277670

**Published:** 2022-11-17

**Authors:** Heung-Bok Kim, John-Paul Bacik, Ruilian Wu, Ramesh K. Jha, Michaeline Hebron, Catherine Triandafillou, Joseph E. McCown, Nam-In Baek, Jeong Han Kim, Young Jae Kim, Celia W. Goulding, Charlie E. M. Strauss, Jurgen G. Schmidt, Gauri S. Shetye, Sungweon Ryoo, Eun-Kyeong Jo, Young Ho Jeon, Li-Wei Hung, Thomas C. Terwilliger, Chang-Yub Kim

**Affiliations:** 1 Bioscience Division, Los Alamos National Laboratory, Los Alamos, New Mexico, United States of America; 2 Hauptman-Woodward Medical Research Institute, Buffalo, New York, United States of America; 3 Georgetown University Medical Center, Washington, D.C., United States of America; 4 Biophysical Sciences Graduate Program, University of Chicago, Chicago, Illinois, United States of America; 5 Array BioPharma Inc., Boulder, Colorado, United States of America; 6 Graduate School of Biotechnology and Department of Oriental Medicine Biotechnology, Kyung-Hee University, Yongin-si, Gyeonggi-do, Republic of Korea; 7 Department of Agricultural Biotechnology, College of Agriculture and Life Sciences, Seoul National University, Seoul, Republic of Korea; 8 Department of Microbiology, Chungnam National University School of Medicine, Daejeon, Republic of Korea; 9 Department of Medical Science, Chungnam National University School of Medicine, Daejeon, Republic of Korea; 10 Infection Control Convergence Research Center, Chungnam National University School of Medicine, Daejeon, Republic of Korea; 11 Department of Molecular Biology and Biochemistry, University of California, Irvine, California, United States of America; 12 Institute for Tuberculosis Research, College of Pharmacy, University of Illinois, Chicago, Illinois, United States of America; 13 Clinical Research Centre, Masan National Tuberculosis Hospital, Changwon-si, Gyeongsangnam-do, Republic of Korea; 14 College of Pharmacy, Korea University, Sejong, Republic of Korea; 15 Physics Division, Los Alamos National Laboratory, Los Alamos, New Mexico, United States of America; 16 New Mexico Consortium, Los Alamos, New Mexico, United States of America; University of East Anglia, UNITED KINGDOM

## Abstract

The ability of *Mycobacterium tuberculosis* (*Mtb*) to persist in its host may enable an evolutionary advantage for drug resistant variants to emerge. A potential strategy to prevent persistence and gain drug efficacy is to directly target the activity of enzymes that are crucial for persistence. We present a method for expedited discovery and structure-based design of lead compounds by targeting the hypoxia-associated enzyme L-alanine dehydrogenase (AlaDH). Biochemical and structural analyses of AlaDH confirmed binding of nucleoside derivatives and showed a site adjacent to the nucleoside binding pocket that can confer specificity to putative inhibitors. Using a combination of dye-ligand affinity chromatography, enzyme kinetics and protein crystallographic studies, we show the development and validation of drug prototypes. Crystal structures of AlaDH-inhibitor complexes with variations at the N6 position of the adenyl-moiety of the inhibitor provide insight into the molecular basis for the specificity of these compounds. We describe a drug-designing pipeline that aims to block *Mtb* to proliferate upon re-oxygenation by specifically blocking NAD accessibility to AlaDH. The collective approach to drug discovery was further evaluated through *in silico* analyses providing additional insight into an efficient drug development strategy that can be further assessed with the incorporation of *in vivo* studies.

## Introduction

The development of a new drug starting from the initial conceptual stage of the project to a marketable product requires extensive research and development including target identification, lead discovery, preclinical development, clinical development, and FDA approval [1], which in practice can take longer than 15 years and cost over $2.5 billion [2, 3]. Since each drug discovery project requires a significant commitment in terms of time and cost, lead compound identification followed by optimization against a selected target is an important step in the early stages of drug discovery that can determine the fate of an entire project [4].

In modern drug discovery, high throughput screening (HTS) of compound libraries has emerged as a standard practice [5, 6]. Particularly with intracellular enzyme targets, most projects start with target-based HTS either by affinity-based or biochemistry-based technologies. The affinity screening approach measures qualitative and quantitative signals based on the physical interactions between target proteins and small-molecule partners. Taking advantage of its capability to handle larger chemical spaces and concentrations with less artifacts [7, 8], this approach is preferred to the biochemistry-based technology, which requires characterization of the proteins and robust reporter assay systems that may limit throughput imposed by testing compounds individually [9, 10].

The process of affinity selection techniques can be carried out using a heterogeneous screening approach that requires immobilization of either macromolecules or small-molecules on a reactive surface, or a homogeneous screening method, in which both macro-molecular targets and small-molecules interact in their native states (with no label) followed by a static or flow-based binding analysis [7]. The homogeneous label-free approaches have advantages of minimal modification of reaction components to study biologically meaningful processes without assay artifacts (e.g. enzyme interactions and ion channel gating) [11, 12]. With these advantages of label-free affinity screening systems, the drug discovery efforts can be performed with enhanced efficiency, cost-effectiveness and high throughput [13]. In this study, we introduce dye-ligand affinity chromatography (DLAC) for label-free affinity HTS.

Dye-ligand affinity chromatography (DLAC) uses dyes that are inexpensive and easy to immobilize as affinity ligands for initial lead compound discovery [14]. One of the most popular triazine dyes, Cibacron Blue F3GA is known to interact with a variety of enzymes such as NAD- and NADP-dependent dehydrogenases, DNA polymerases, kinases, glucose oxidase, lysozyme, albumin, catalases, and plasma proteins [15–20]. Resembling common nucleotides with composition of an anthraquinone and a terminal phenylsulfonate ring, of which chemical structures are similar to those of nicotinamide and adenine respectively, the molecule also contains two internal aromatic rings that mimic ribofuranose rings, while the two anionic sulfonate groups may behave similar to phosphates [21, 22]. The F3GA dye is known to interact through the nucleoside-interfacing site of many nucleoside-binding proteins, which account for almost half of the entire enzyme population in any organism [23]. We recognized that this dye could be used in ligand screening based on ligand-specific displacement of a protein at the dye-binding site [24, 25].

Tuberculosis (TB) is one of the top ten most prevalent causes of death worldwide, with 95% of TB deaths occurring in lower income countries [26]. The emergence and rapid spread of multidrug-resistant (MDR)-TB and extensively drug-resistant (XDR)-TB are major challenges to the global control of TB [27]. In the infected lungs of TB patients, the bacteria can shift to a non-replicating persistent phase (NRP) which is associated with hypoxia [28, 29]. Recently, it has been proposed that the antimicrobial resistance may develop in *Mycobacterium tuberculosis* (*Mtb*) during this NRP period [30–32].

*Mtb* L-alanine dehydrogenase (AlaDH) catalyses the reversible reaction in conversion of L-alanine to pyruvate concurrent with an NAD-dependent reaction [S1 Scheme in S1 File, 33–35], and is associated with peptidoglycan synthesis and maintenance of an NAD pool under stress conditions [36, 37]. Based on the studies revealing that (a) the gene encoding AlaDH (Rv2780) is up-regulated in *Mtb* in response to hypoxia, (b) AlaDH gene knockout mutant of *Mtb* resulted in a significant lag in the resumption of growth after re-oxygenation, and (c) the reactivated AlaDH mutant had an altered NADH/NAD ratio [35, 36, 38, 39], AlaDH is proposed to maintain the optimal NADH/NAD ratio during anaerobiosis to prepare for regrowth by responding early to re-oxygenation [39]. From these data, the AlaDH is recognized as one of the top potential drug targets against *Mtb* persistence [40–42].

We previously carried out a genome-wide survey of nucleotide triphosphate (NTP) binding proteins in *Mtb* through structurally guided computational approaches and identified a set of 1768 such proteins [43]. Applying the independent DLAC method to this study, 47 proteins including AlaDH were identified and characterized by nucleoside binding features. The DLAC approach could be also applied to enhancement of crystallization by sorting out the interacting nucleoside ligands of AlaDH and other *Mtb* proteins [25].

In this report, based on our studies on AlaDH using the DLAC technique, we described another application of DLAC for label-free affinity screening, and demonstrated its usability for drug discovery and development. We have implemented a combination of approaches including DLAC, X-ray crystallization, compound design and synthesis, enzyme kinetics and docking simulations to identify a new class of compounds that target *Mtb* AlaDH. Here, we manifested an effective combination of these approaches for drug lead identification and optimization, and addressed issues of specificity that are critical to the design of inhibitors that target nucleoside binding regions of dehydrogenase proteins.

## Materials and methods

### Cloning of *Mtb* L-alanine dehydrogenase and aldehyde dehydrogenase genes

*Mtb* L-alanine dehydrogenase gene (Rv2780) was amplified by PCR from *Mtb* H37Rv genomic DNA with Pfu polymerase, using the 5’ *NcoI* primer, 5’-AGATATACCATGG + (N-terminal 20 bases of Rv2780 sequence) -3’, and the 3’ *NotI* primer, 5’-AATTCGCGGCCGC + (C- terminal 20 bases of Rv2780 sequence) -3’. The underlined bases represent the *NcoI* and *NotI* sites, respectively. The PCR amplicon was digested with *NcoI* and *NotI* restriction endonucleases, and purified using Qiaquick PCR spin column. The product was ligated into a pETM-11 vector using T4 DNA ligase. The expression vector of *Mtb* aldehyde dehydrogenase (Rv0223c) was constructed by following the protocol described previously [24]. The insertion of these two genes into the plasmids was confirmed by DNA sequencing.

### Point mutations on *Mtb* L-alanine dehydrogenase

Point mutated AlaDH gene of L225A or L249A was generated by using QuickChange Lightning Site-Directed Mutagenesis Kit (Agilent Technologies). Each point mutated plasmid was generated from pETM-11 plasmid containing AlaDH gene using primers 5’ -TCATCGGCCTACGAGGCGGAGGGTGCCGTC AAA- 3’ and 5’ -TTTGACGGCACCCTCCGCCTCGTAGGCCGATGA- 3’ (for L225A) or 5’–GCCAAG GCACCCAAAGCGGTCTCGAATTCACTT- 3’ and 5’–AAGTGAATTCGAGAC CGC TTTGGGTGCCTTGGC- 3’ (for L249A)

### L-alanine dehydrogenase and aldehyde dehydrogenase expression and purification

Each different dehydrogenase clone was transformed into *E*. *coli*. BL21(DE3) cells to express. Cells were grown at 37°C in LB medium containing 100 μg/ml ampicillin, induced with 1 mM IPTG when OD600 reached 1.0, and grown at 25°C overnight in a shaking incubator set at 250 rpm. The cells were harvested by centrifugation and the cell pellets were stored at -80°C. The expression of each protein was confirmed by SDS-PAGE [44].

For purification of the dehydrogenases, frozen cells were thawed on ice and resuspended in lysis buffer (20 mM Tris-HCl pH 8.0, 200 mM NaCl, 1 mM PMSF, 1 mg/ml DNase, 1 mM MgCl_2_). Lysates were sonicated and then centrifuged with 3,000 g at 4°C for 30 min. The supernatant was filtered through a 0.45 μm pore membrane and loaded on a 5 ml Ni-NTA superflow affinity column. After being washed with buffer A (20 mM Tris-HCl pH 8.0, 200 mM NaCl), the target protein was eluted by buffer B (buffer A plus 500 mM Imidazole). To remove the contaminants, eluted fractions were further purified on a Superdex-75 gel filtration column using buffer C (10 mM Tris-HCl pH 8.0, 150 mM NaCl, and 1 mM DTT). The peak fractions (monitored at OD_280_) were analyzed by SDS-PAGE and centrifugal concentrator was used to concentrate the pooled protein fractions to 10–15 mg/ml, as measured by Bradford reagent. Protein purity was confirmed by SDS-PAGE.

### Screening of drug compounds interaction with L-alanine dehydrogenase by dye-ligand affinity chromatography

To screen multiple compounds binding to L-alanine dehydrogenase, we followed a modified version of the protocol described in Kim *et al*. [24]. Briefly, in each well of 96-well multiscreen plate with a filter at the bottom, 20 μg of purified L-alanine dehydrogenase was loaded on to 25 μl of Cibacron blue F3GA dye-resin equilibrated with column buffer (CB; 50 mM potassium phosphate, pH 7.5, 1 mM MgCl_2_ and 2 mM DTT). After gentle vortex to bind protein to the resin for 1 hr at 4°C, unbound protein was removed by 5 times washing with 250 μl of CB. The protein was eluted after 1 hour of incubation with 30 μl of each compound (at 1 mM) from 36 anti-TB compounds (shown in S1 Table and S1 Fig in **S1 File**.). In washing and eluting processes, the air-suction from the bottom of the 96-well plate of Bio-robot 8000 was used before adding a new solution to each well. Aliquots of eluate fractions were diluted with 1:1 volume ratio with 2x SDS sample buffer, and 15 μl was loaded on 10% SDS-PAGE. For quantitative evaluation of the interaction of each compound with AlaDH, the gel-bands stained by Coomassie Brilliant Blue G-250 were scanned by densitometry, and the AlaDH amount eluted by each compound was calculated relative to the protein amount eluted by N6-methyl ado. The gel band measurements were carried out in triplicate and averaged.

### Inhibition assays for L-alanine dehydrogenase and aldehyde dehydrogenase enzymes

The inhibition assays of both AlaDH and AldDH were performed monitoring NADH production by measuring OD340 ([NADH]_OD340_) with Tecan Infinite 200 microplate reader in a total volume of 0.1 ml of enzyme reactions. All assays were carried out at room temperature at least in triplicate.

**(i) AlaDH inhibition assay using synthesized compounds** In the reaction mixture of 50 mM Tris-HCl pH 7.5, 17 mM L-alanine and 1 mM NAD with additional 1 mM each inhibitor compound or without it as a control, 2 μl of 15 μM of AlaDH was added to start the reaction which was monitored for NADH production by OD340 every minute for 30 min.

**(ii) AldDH inhibition assay using synthesized compounds** In the reaction mixture of 50 mM Na-phosphate pH 7.4, 10 mM propionaldehyde and 1 mM NAD with additional 1 mM each inhibitor compound or without it as a control, 7 μl of 23 μM of AldDH was added to start the reaction which was monitored for NADH production by OD340 every minute for 30 min.

**(iii) *K***_***m***_ **of NAD and *K***_***i***_ **measurement for inhibitor compounds of AlaDH** In a buffered solution of 25 mM Tris-HCl pH 7.5, 7.5 mM L-alanine, and eight different concentrations from 0.1 up to 1.6 mM NAD with none or three separate concentrations (as indicated in Fig 3B) of inhibitor compound for each reaction, the reaction was initiated upon the addition of 0.3 μM AlaDH and monitored for 5 min. The *K*_*m*_ of NAD and *K*_*i*_ value of individual compound was calculated in competitive inhibition mode based on the profile of Lineweaver-Burk plots using Prism 9 software (GraphPad Software) with the data attained from experiments in triplicate.

### Co-crystallization and X-ray data collection of L-alanine dehydrogenase with N6-methyl adenosine and N6-isobutyl adenosine

L-alanine dehydrogenase was co-crystallized with N6-methyl adenosine (ado) or N6-isobutyl adenosine (ado), respectively. For initial screening, hanging drops (1 μl protein-ligand solution + 1 μl reservoir solution) were set up in 24-well plates using crystal screen 1 and 2 (Hampton Research). If small crystals were found, crystallization conditions were optimized, if possible, by fine-tuning each component until crystals with dimensions of at least 50 μm x 50 μm x 50 μm were obtained. L-alanine dehydrogenase was mixed with N6-methyl ado or N6-isobutyl ado at a molar ratio of 1:5 (protein: ligand) and incubated on ice for 30 min prior to set-up crystallization. For x-ray data collection, a minimum of five protein crystals grown with each compound were selected based on size and morphology, harvested and flash-cooled in liquid N_2_, with the addition of 10% glycerol in the buffer as cryo-protectant. Monochromatic datasets were collected at the beam lines 5.0.1 and 5.0.2 at the Advanced Light Source (ALS). All datasets were processed with the HKL2000 program suite [45]. The detailed data collection and refinement statistics are listed in Table 1.

**Table 1:** Data collection and refinement statistics.

	Rv2780 + N6-methyl adenosine (PDB:4LMP)	Rv2780 + N6-isobutyl adenosine (PDB: 6O7F)
*Wavelength (Å)*	0.9774	0.9774
*Resolution range*	46.52–1.95 (1.98–1.95)	48.33–2.30 (2.34–2.30)
*Space group*	R 3 2: H	R 3 2: H
*Unit cell*	89.730 89.730 290.401 90 90 120	88.423 88.423 289.991 90 90 120
*Total reflections*	194075	142116
*Unique reflections*	33325 (1602)	19578 (983)
*Multiplicity*	5.8 (4.1)	7.3 (6.7)
*Completeness (%)*	100.0 (99.9)	98.70 (98.60)
*Mean I/sigma(I)*	22.84 (2.23)	18.63 (1.19)
*Wilson B-factor*	23.99	32.73
*R-merge*	0.072 (0.478)	0.097 (1.206)
*Reflections in refinement*	32209 (1412)	17109 (442)
*Reflections used for R-free*	1933 (82)	1031 (26)
*R-work*	0.1853 (0.2492)	0.1897 (0.2435)
*R-free*	0.2248 (0.2771)	0.2338 (0.2676)
*# of non-hydrogen atoms*	3011	2861
*macromolecules*	2741	2727
*ligands*	59	23
*solvent*	211	111
*Protein residues*	371	371
*RMS(bonds)*	0.008	0.005
*RMS(angles)*	1.18	0.76
*Ramachandran favored (%)*	98.37	98.10
*Ramachandran allowed (%)*	1.63	1.90
*Ramachandran outliers (%)*	0.00	0.00
*Rotamer outliers (%)*	3.23	3.61
*Clashscore*	3.75	4.91
*Average B-factor*	27.03	47.54
*macromolecules*	26.52	48.02
*ligands*	33.68	32.89
*solvent*	31.88	38.77

Statistics for the highest-resolution shell are shown in parentheses.

## Results

### Targeting *Mtb* AlaDH using dye-ligand affinity chromatography and structure-function studies

**Fig 1:**
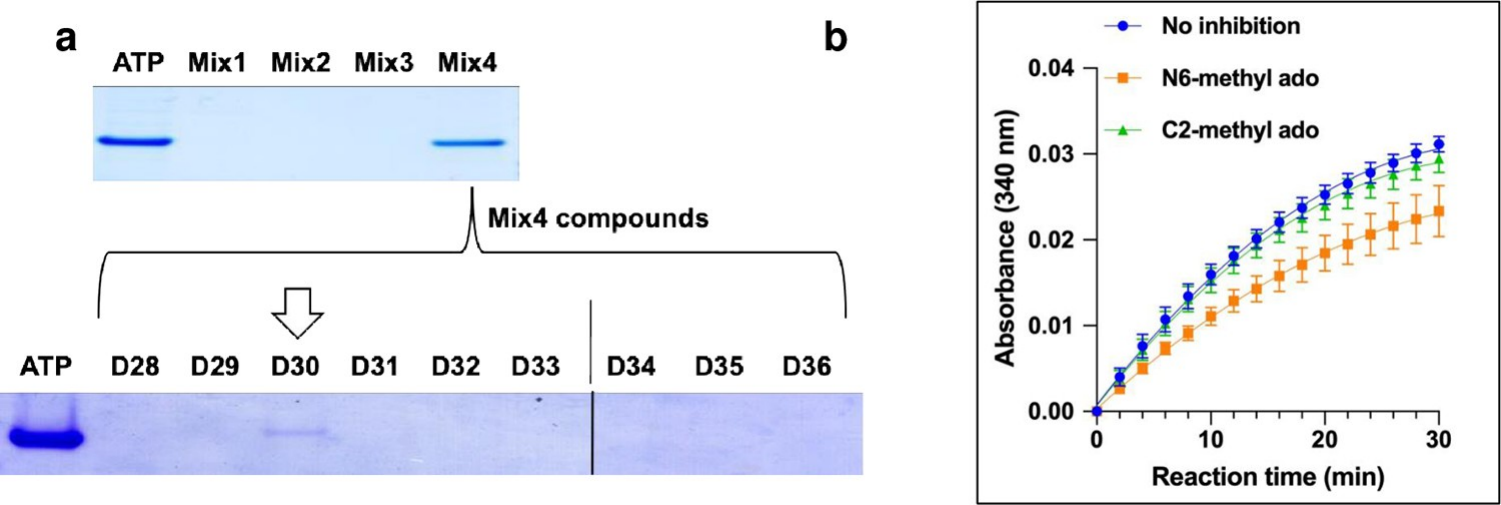
Drug hit identification. (a) DLAC screening tests reveal N6-methyl adenosine (D30) as a putative hit component against AlaDH. D28, C2-methyl adenosine; D29, cyclohexyl 2-[[5-(3,4-dimethoxyphenyl)-1,3,4-oxadiazol-2-yl]sulfanyl]acetate; D30, N6-methyl adenosine; D31, 4-[[5-(4-methylphenyl)-1,3,4-oxadiazol-2-yl]sulfanyl]but-2-ynyl furan-2-carboxylate; D32, N-[4-(4-butanoylpiperazin-1-yl)-3-chlorophenyl]-5-nitrofuran-2-carboxamide; D33, 5-(1,3-benzodioxol-5-yl)-N-(5-chloro-2-hydroxyphenyl)-7-(trifluoromethyl)-1,5,6,7-tetrahydro pyrazolo[1,5-a]pyrimidine-2-carboxamide; D34, 5-(4-bromophenyl)-N-[(1,5-dimethylpyrazol-4-yl)methyl]-7-(trifluoromethyl)-1,5,6,7-tetrahydropyrazolo[1,5-a]pyrimidine-3-carboxamide; D35, 4-methyl-N-(5-pyridin-4-yl-1,3,4-oxadiazol-2-yl)benzamide; D36, benzyl (2S)-4-methyl-2-[[4-[(3-methylphenyl) carbamoyl]-1H-imidazole-5-carbonyl]amino]pentanoate. The gels are cropped from the full-length gels which are presented in S2a and S2c Fig in **S1 File**. ATP is used as a positive control based on our previous data [25], where NADH and ATP (but not NAD) bound strongly with this assay (b) Inhibition effect of N6-methyl adenosine is confirmed using enzyme kinetic studies.

Based on our previous studies for high throughput analysis of compounds interacting with the AlaDH enzyme, which was bound to the F3GA dye resin [31, 32], we first performed screening of a pool of 36 nucleoside-derivative anti-TB compounds (S1 Fig in **S1 File**.) that are selected and supplied by the Southern Research Institute based on their whole cell screening results that revealed minimum 85% of *Mtb* growth inhibition [46, 47]. Fig 1A shows interaction analyses of compounds against the target protein, AlaDH using DLAC analysis of multi-drug mixtures followed by DLAC analyses of individual compounds from mixtures that showed positive results. DLAC analyses of four drug mixtures (Mix1- Mix4) that contain 8–10 anti-TB drug compounds each (for a total of 36 compounds listed in S1 Table in **S1 File** and their chemical structures in S1 Fig in **S1 File**), showed that only Mix4 eluted the target AlaDH enzyme along with the positive control, ATP (Fig 1A). To assess which drug(s) in the Mix4 elute(s) AlaDH, the 9 individual drugs (D28 –D36 of S1 Table and S1d Fig in **S1 File**) were tested individually using the DLAC assay. Analysis of the elutes showed that only D30 (N6-methyl adenosine) displaced the F3GA dye from AlaDH, confirming the feasibility of this approach and highlighting this anti-TB compound as a new potential drug lead against AlaDH.

Based on the DLAC interaction assay results, using the AlaDH reaction to convert L-alanine to pyruvate by using NAD^+^ as cofactor (S1 Scheme in **S1 File**), we carried out an activity inhibition assay for the D30 compound (N6-methyl adenosine) as well as with the structurally similar D28 compound (C2-methyl adenosine), which did not show displacement of AlaDH from the dye resin (Fig 1A and 1B). Compound D30 reduced the initial velocity (vi) by 33% to 1.231x10^-3^ [NADH]_OD340_/min of the AlaDH reaction from the vi value of the AlaDH reaction with no inhibition, 1.842x10^-3^ [NADH]_OD340_/min. However, the inhibitory effect of D28 was significantly lower (4%) as calculated from its vi, 1.769 x10^-3^ [NADH]_OD340_/min. We next examined the effects of N6-methyl adenosine (ado) in murine primary bone marrow-derived macrophages (BMDMs) infected with *Mtb* (moi = 1). As shown in S3 Fig in **S1 File**, intracellular growth of *Mtb* was significantly decreased in BMDMs by treatment with N6-methyl ado. The minimum inhibitory concentration (MIC) test of this compound turned out to be ~10 μg/ml as revealed in S4 Fig in **S1 File**. These data suggest that the N6-methyl group plays an important role in the drug potency in comparison with a natural metabolite, adenosine.

**Fig 2:**
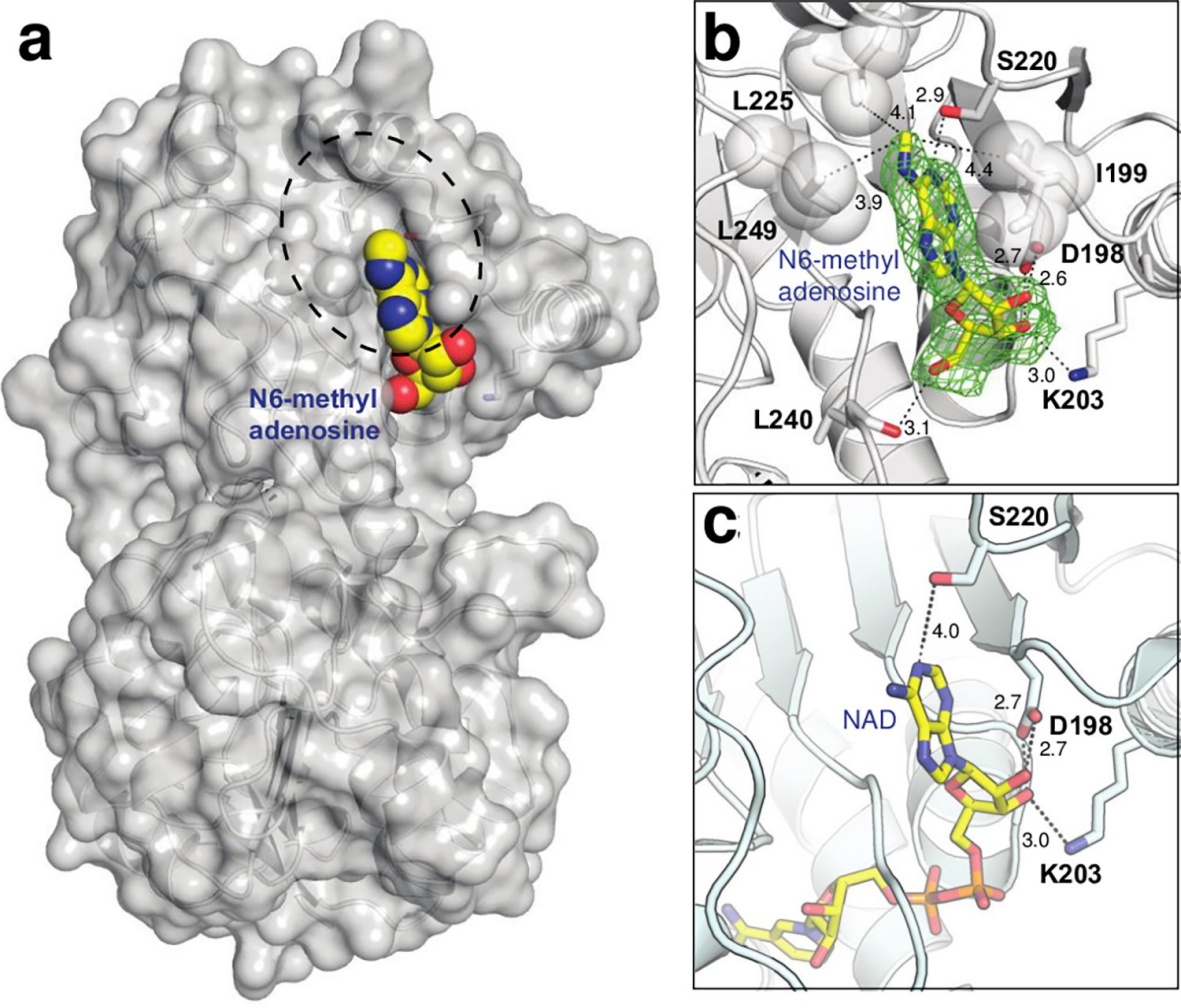
Crystallographic AlaDH structure. (a) Crystal structure of AlaDH bound to N6-methyl adenosine. Dashed circle demarcates the hydrophobic binding groove. (b) Close-up view of the nucleoside/inhibitor binding site. The electron density map is a polder omit map [48] at 3σ. (c) Crystal structure of AlaDH bound to NAD (PDB code 2VHX, [34]). Atom colors are red (oxygen), blue (nitrogen), yellow (ligand carbons) and gray (protein carbons). Hydrogen bonds are shown as dashed lines with distances in angstroms.

To further investigate the interaction between compound D30 and AlaDH, we determined a crystal structure of AlaDH complexed with N6-methyl ado at 1.95 Å resolution (PDB code, 4LMP) (Fig 2A). The structure showed that N6-methyl ado binds in the same region of AlaDH as the natural ligand NAD and forms many of the same interactions (Fig 2B and 2C). While the N6-methyl moiety appears to form favorable hydrophobic interactions with Leu225 and Leu249, other protein residues (Asp198, Lys203, Ser220) are also involved in binding, including a main chain interaction with Leu240. The nature of the compound binding thus appears to make use of the protein nucleoside binding site as well as a natural hydrophobic region of the protein, and we do not observe significant structural or conformational changes. Since N6-methyl ado is involved in a variety of natural biological processes we sought to evaluate different modifications that may affect specificity towards AlaDH.

### Optimization of compounds targeting *Mtb* AlaDH through rational approaches

Examining the binding of D30 to AlaDH, we noted that the hydrophobic groove formed by residues Leu225 and Leu249 that interact with N6-methyl group continues with the side chains of hydrophobic residues Val250 and Leu254. This suggested that adenosine analogues with hydrophobic groups extending further from the N6 position might lead to stronger inhibitors against AlaDH and greater specificity for this *Mtb* enzyme. We therefore designed second-generation compounds that extend hydrophobic interactions further along the hydrophobic groove in AlaDH. Aromatic ring systems were excluded to avoid competition with the adenine binding site. Alkyl (ethyl-, propyl- and isobutyl-), as well as, alkylester (acetyl- and isobutanoyl-esters) groups were introduced at position N6 of the adenosine to evaluate the relative contributions of hydrophobicity and potential bridged hydrogen’s bonding to adjacent AlaDH residues. We also hypothesized that the extended alkyl chains might increase steric interference and reduce binding to other adenosine binding enzymes. N-acyl and N-alkyl adenosines were prepared according to S2 and S3 Schemes in **S1 File**, respectively [49]. Using these protocols we synthesized five N6-methyl ado derivatives (N6-ethyl, N6-propyl, N6-isobutyl, N6-acetyl and N6-isobutyoyl ados) as shown in S5 Fig in **S1 File**. The chemical identity of each newly synthesized compound were confirmed by analyses of melting point, specific rotation, 1H NMR, 13C NMR, infrared radiation (IR) and high-resolution mass spectral (HRMS) data (S3 Method in **S1 File**).

**Fig 3:**
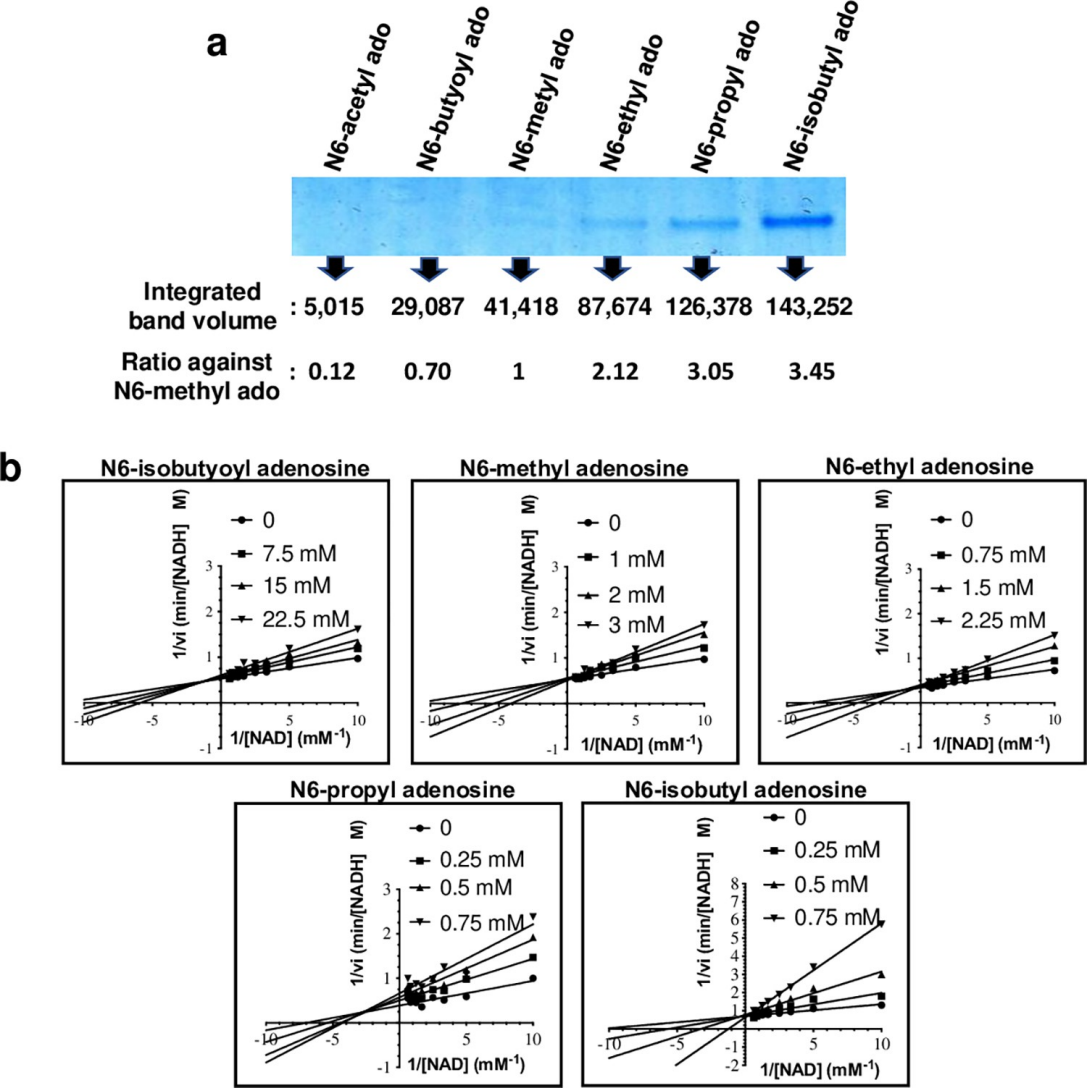
Characterization of synthesized compounds. (a) Analysis of six synthesized adenosine analogue compounds (including the lead compound, N6-methyl adenosine) with AlaDH by DLAC and densitometry analyses. The gel is cropped from the full-length gel that is presented in S6 Fig in **S1 File**. (b) Lineweaver-Burk plots to calculate the *K*_*i*_ of five compounds based on their activity inhibition at indicated concentrations against AlaDH.

The compounds were evaluated with the DLAC interaction assay, with quantification of AlaDH elution by each compound using SDS gel densitometry (Fig 3A). Both N6-acetyl ado and N6-butyoyl ado eluted less AlaDH than N6-methyl ado, showing 12% and 70% of eluted protein amount relative to N6-methyl ado. In contrast, N6-ethyl ado, N6-propyl ado and N6-isobutyl ado showed significantly higher amounts of eluted AlaDH with over two to threefold (2.12, 3.05 and 3.45 respectively) eluted AlaDH compared to N6-methyl ado.

The relative AlaDH amounts eluted from the dye resin by compounds with varying substituents suggests that the carbonyl groups in acetyl- and butanoyl-groups at N6 of adenosine caused the decrease in affinity of these compounds to AlaDH compared to N6-methyl ado, possibly by causing an unfavorable structural clash with the Ser220 side chain. To this extent, Ser220 is the only amino acid that appears to be in close enough proximity to cause such interference, which is otherwise made up of a pocket of hydrophobic residues (Fig 2B). Although the additional methyl in N6-butanoyl ado likely compensates for some of the affinity reduction caused by the carbonyl-group, it was however promising that as we predicted, lengthening the hydrophobic groups at N6 with ethyl and propyl chains for enhanced interaction with the hydrophobic groove in AlaDH actually promoted more favorable interactions with AlaDH compared to the N6-methyl ado. Even more striking was that the branched N6-isobutyl group showed greatly enhanced affinity with AlaDH, which suggests a better fit between the moiety and the hydrophobic groove of the enzyme.

While these results obtained by DLAC distinguish somewhat quantitatively the affinity differences among the rationally designed compounds, in order to further validate these findings, we tested the AlaDH inhibitory effect of the five newly designed compounds along with the original lead compound on the activity of AlaDH. Fig 3B shows Lineweaver-Burk plots for each compound and Table 2 shows the calculated *K*_*m*_ and *K*_*i*_ values. Since N6-acetyl ado did not reveal any inhibitory effect, the values of this compound were not measured. Consistent with our results with DLAC, several of the compounds showed larger *K*_*m*_ values for the substrate NAD than the original hit compound N6-methyl ado (*K*_*m*_ = 0.082 mM) like 0.090 mM (N6-ethyl ado), 0.124 mM (N6-propyl ado), and 0.163 mM (N6-isobutyl ado) indicating that each compound reduced the accessibility of the substrate NAD to AlaDH by competing for its binding site, and lower *K*_*i*_ values than N6-methyl ado (*K*_*i*_ = 0.86 mM), including N6-ethyl ado (*K*_*i*_ = 0.52 mM), N6-propyl ado (*K*_*i*_ = 0.11 mM), and N6-isobutyl ado (*K*_*i*_ = 0.08 mM).

**Table 2:** *K*_*m*_ of NAD and inhibition constants (*K*_*i*_) of adenosine analogue compounds for *Mycobacterium tuberculosis* L-alanine dehydrogenase.

Compounds	*K*_*m*_ in mM (S.D.*)	*K*_*i*_ in mM (S.D.*)
N6-isobutyoyl adenosine	0.078 (0.013)	3.714 (0.641)
N6-methyl adenosine	0.082 (0.016)	0.861 (0.300)
N6-ethyl adenosine	0.090 (0.023)	0.521 (0.150)
N6-propyl adenosine.	0.124 (0.038)	0.113 (0.022)
N6-isobutyl adenosine	0.163 (0.034)	0.079 (0.015)

*S.*D*.: Standard Deviation

The compounds N6-propyl ado and N6-isobutyl ado each had increased inhibitory effects compared to the hit compound, N6-methyl ado, indicating that the compound design whereby additional methyl groups introduced at N6 is effective for the purpose of lead optimization. From the inhibition profiles of all the compounds we assayed against the target protein, the results appear to correspond to the elution profile of AlaDH by the DLAC assay, as they show the same rank-order as well as similar relative levels of inhibition (S7 Fig in **S1 File**). Moreover, the two assay methods are completely independent of each other in measuring the affinity of compounds to the target protein or the inhibition of each compound on the target’s enzymatic activity, respectively.

**Fig 4:**
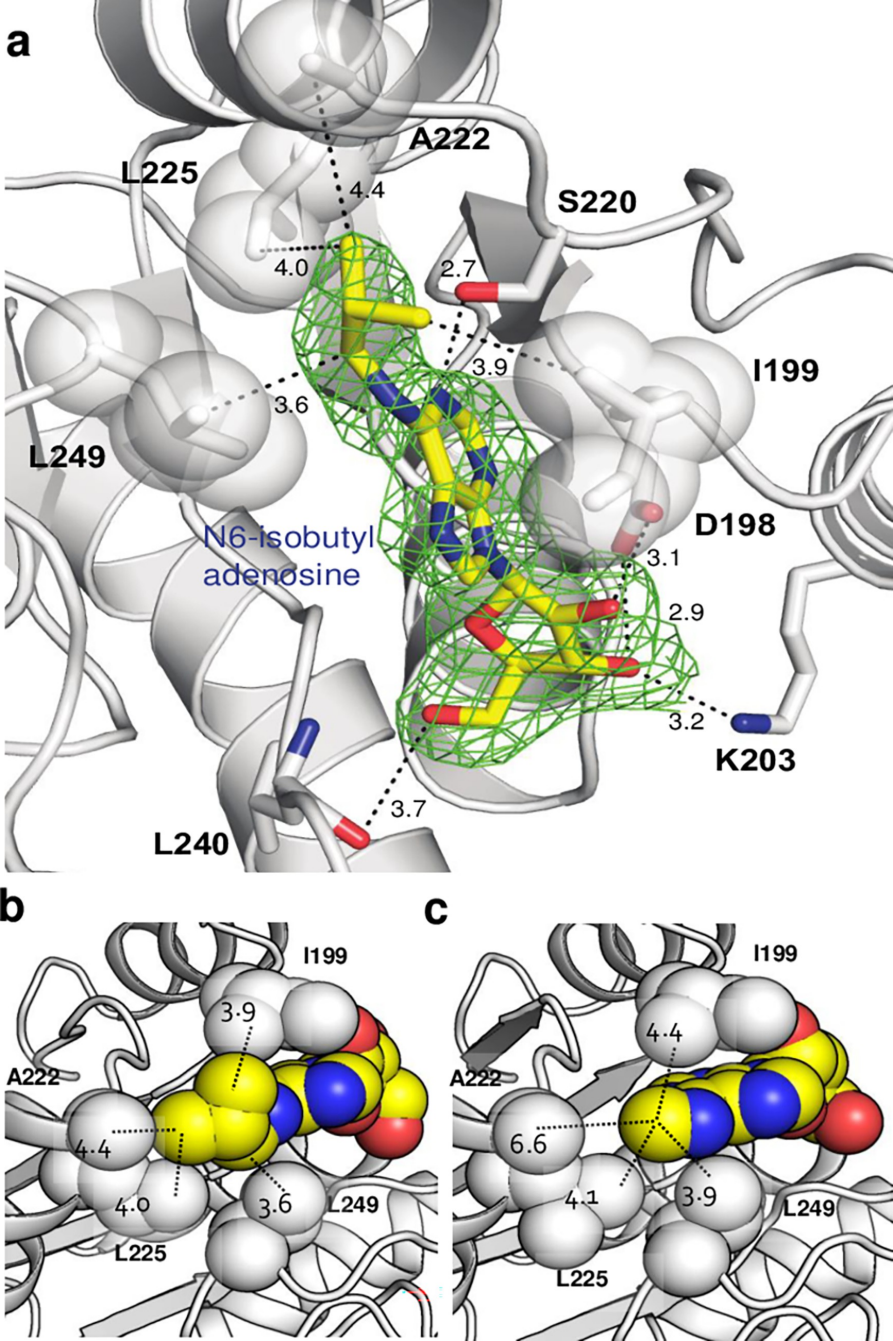
AlaDH crystal structure in complex with adenosine-based inhibitors. (a) N6-isobutyl adenosine inhibitor binding site showing hydrophobic interactions and hydrogen bonds. Electron density is shown as polder omit map at 3σ [48]. (b) Crystallographic structure bound to N6-isobutyl adenosine and (c) N6-methyl adenosine. Dashed lines show closest interatomic distances between the N6-methyl or N6-isobutyl moieties to surrounding hydrophobic residues. It can be seen that each distance for the N6-methyl moiety is longer than the corresponding nearest distance to the N6-isobutyl moiety (6.6 *vs*. 4.4 Å for A222; 4.1 *vs*. 4.0 Å for L225, 3.9 vs. 3.6 Å for L249; 4.4 vs. 3.9 Å for I199).

To examine our compound design by inhibition mode analysis, we solved the co-crystal structure of AlaDH with N6-isobutyl ado (2.30 Å) as shown in Fig 4A (PDB code, 6O7F). The N6-isobutyl ado is observed at the nucleoside binding site, forming several of the same interactions as the structures bound to NAD and N6-methyl adenosine. Importantly, when comparing the two inhibitor bound structures, the distances between the closest carbon atoms of surrounding residues I199, A222, L225 and L249 are shortened for the N6-isobutyl structure (Fig 4B and 4C). Based on these observations, the enhanced affinity of N6-isobutyl ado by ~3.5 fold in DLAC assay and its increased inhibition by about 10-fold from *K*_*i*_ compared to the original lead compound, are likely derived from the stronger hydrophobic interaction between the isobutyl group of the compound and the hydrophobic residues that surround the isobutyl moiety. These results combined with the structure analysis also suggest that it may be possible to further optimize these compounds by extending the isobutyl group with additional hydrophobic chemical groups.

### Evaluation of drug specificity through *in vitro* and *in silico* analyses

To further evaluate the interaction between the isobutyl group of N6-isobutyl ado and residues close to its hydrophobic moiety (Fig 4B), we generated point-mutations (L225A or L249A) for AlaDH and analyzed their influence on inhibition (S8 Fig in **S1 File**). With no N6-isobutyl ado added, both L225A and L249A AlaDHs reveal similar enzyme kinetic profiles to wild-type AlaDH with vi of each, 1.925 x10^-3^ [NADH]_OD340_/min (L225A), 2.020 x10^-3^ [NADH]_OD340_/min (L249A) and 1.922 x10^-3^ [NADH]_OD340_/min (wild-type) indicating little or no influence of mutations on enzyme activity. However, after adding N6-isobutyl ado in the reaction, the inhibition effects by N6-isobutyl ado for both L225A and L249A AlaDHs were reduced relative to the wild-type by showing vi of 0.821 x10^-3^ [NADH]_OD340_/min (L225A) and of 1.007 x10^-3^ [NADH]_OD340_/min (L249A), while the vi of wild-type AlaDH is demonstrably lower at 0.734 x10^-3^ [NADH]_OD340_/min. This result indicates that both Leu225 and Leu249 are interacting with N6-isobutyl ado and involved in its inhibition mode, but that Leu249 contributes more to this inhibition. Future work will also examine through mutagenesis how other residues in the groove (I199, A222) contribute to binding of the inhibitors.

To gain insight into the specificity of these compounds with other NAD-binding enzymes, we carried out an activity assay for a different enzyme, *Mtb* aldehyde dehydrogenase (Rv0223c, AldDH) that also uses NAD as a cofactor [24]. We found no inhibition of AldDH activity by N6-isobutyl ado, and also none by N6-methyl ado (S9a Fig in **S1 File**). Supporting these observations, inspection of a previously determined AldDH crystal structure solved with NAD (S9b Fig in **S1 File** having the PDB code, 3B4W) also indicates that AldDH lacks enough room to accommodate the additional branch at N6 on adenosine with a methyl or larger groups. To evaluate the specificity of N6-isobutyl ado with other dehydrogenases, we carried out ligand-docking simulations using the Cambridge Crystallographic Data Centre (CCDC) Gold for ligand fitting [50] and a knowledge-based scoring function (DrugScore eXtended, DSX) [51]. We compared the interaction score-potentials of N6-isobutyl ado docked in the *Mtb* AlaDH structure and N6-isobutyl NAD docked to four human dehydrogenase crystal structures of human malate DH 2, human hydroxyacyl CoA DH, human sorbitol DH, and human 15-hydroxyprostaglandin DH type1 (S9c Fig in **S1 File**). The docking analyses show favorable interactions of both the N6-isobutyl and the adenosine moieties with *Mtb* AlaDH. Conversely, unfavorable interactions of the isobutyl chain of the N6-isobutyl NAD were identified for each of the human DH. Moreover, similar results were obtained for nine other crystal structures of dehydrogenases (S10 Fig in **S1 File**), and these results were further supported by no noticeable inhibition of N6-methyl ado and N6-isobutyl ado from the activity assays against human malate DH 2 (S9d Fig in **S1 File**), also supporting the specificity of our compound.

## Discussion

We have applied a combination of techniques for analysis of protein-ligand binding interactions [24, 43] and inhibition effects as well as using these results for enhancement of crystallization by co-crystallization with identified ligands [25]. The results from the DLAC correlated to the enzyme inhibition studies, which were then further corroborated by structural analysis. This suggests that the binding studies alone are a promising strategy for early-phase determination of the druggability of a particular target, which could facilitate high-throughput drug discovery and optimization strategies. Conducting the DLAC assay for drug lead identification in 96- (or 384-) well format also effectively lends itself to scaling in HTS, with other modifications that could increase throughput and feasibility such as; (i) automation of loading of protein bound dye-resin and elution of protein using library compounds delivered by a liquid-handling robot; (ii) detecting the eluted protein with high throughput 96- (or 384-) well format Bradford or similar assay system; (iii) use of thermal denaturation studies to determine what effect inhibitors have on protein stability (i.e. differential scanning fluorimetry) that can also be readily assessed using HTS [52, 53].

The DLAC assay demonstrated in this report can likely be implemented towards other nucleos(t)ide binding drug targets. Nucleos(t)ide binding proteins are predominantly vital to the cell, being involved in cell signaling, metabolism of nucleotides, amino acids, carbohydrates, and lipids. The candidate proteins that can be targeted using this method will cover a wide range of essential proteins including dehydrogenases, reductases, and kinases. These proteins have been well recognized as drug target classes in major worldwide diseases such as malaria, Alzheimer’s disease, cancer, and inflammation [54–63].

As highly drug-resistant *Mtb* strains continue to evolve, the risk of losing control over possible outbreaks becomes greater [64, 65]. With the knowledge that AlaDH participates directly in *Mtb* persistence [39], which provides an evolutionary opportunity to allow the emergence of resistant strains [66, 67], the Sriram group pioneered the development of inhibitors targeting AlaDH by application of virtual screening based on the crystal structures of AlaDH in complex with NAD (PDB code, 2VHW) and with N6-methyl adenosine (PDB code, 4LMP) that we determined as shown in Fig 2 [41, 42, 68–70]. Their compounds displayed anti-TB activity against both active and nutrient-starved dormant *Mtb* cells with MIC range from 1.53 to 60.38 μM, and the 2-ethyl-N-phenethyl-5,6,7,8-tetrahydrobenzo[4,5]thieno[2,3-d]pyridin-4-amine inhibited AlaDH with IC50 of 1.82±0.42 μM and revealed 2.7 log reduction of dormant *Mtb* cells at 10 μg/ml by showing more potency than isoniazid and rifampicin [69]. While promising, further development and optimization of the compounds to enhance their potency and specificity will be needed to improve their effectiveness as potential therapeutics.

In this report, we present an early part of a drug development pipeline showing hit identification process using our DLAC approach, and its subsequent optimization by rational drug design based on interactions with AlaDH. After structural identification of a N6-methyl adenosine inhibition mode that blocks NAD by occupying the interaction site of its adenosine component, an optimization process was carried out based on the observation that the N6-methyl moiety of the lead faces along the extended hydrophobic groove structure. A lead compound with *K*_*i*_ of 860 μM was optimized by designing adenosine analogue compounds with extensions at the N6 position to enhance the hydrophobic interactions, and by confirmation of affinity and inhibition improvement through the DLAC and activity assays. In this optimization, the *K*_*i*_ was improved over 10-fold, yielding the compound N6-isobutyl ado with a *K*_*i*_ of 80 μM. From the co-crystal structure of N6-isobutyl ado with AlaDH, the shortened atomic distances between N6-isobutyl and surrounding hydrophobic AlaDH residues (Fig 4) indicated that we improved the ligand-protein interaction through our design that resulted in the lower measured *K*_*i*_ value.

Through this optimization cycle, we identified the site that confers specificity as being located adjacent to the N6 portion of the hit compound (or NAD). This observation was corroborated by comparison to other DHs with docking and inhibition analyses. This site may be useful in rational design of next generation inhibitors of AlaDH to block its ability to supply energy to *Mtb* under persistent state and to cause relapse of *Mtb* manifestation with re-oxygenation.

Drug development for DHs may include (i) direct targeting against the cofactor NAD(P) binding site, and (ii) the design of enzyme inhibitors (or activators) based on the target DH structure [71]. Since many DHs use NAD(P) as a substrate and bind them using a highly conserved structural motif, despite its theoretical possibility, designing such inhibitors specific to a single enzyme remains challenging, and the physiological relevance remains an important aspect to be studied [72, 73]. Our combination of approaches targeting a specificity-conferring site of *Mtb* AlaDH has worked towards addressing this limitation and enlightened the direction of drug discovery against other DH targets and of subsequent iterations of drug development aimed at *Mtb* that would include *in vivo* studies.

## Supporting information

S1 File (DOCX)
