# Supplementary material for: Label-free affinity screening, design and synthesis of inhibitors targeting the Mycobacterium tuberculosis L-alanine dehydrogenase
Source: PLoS One. 2022 Nov 17;17(11):e0277670. doi: 10.1371/journal.pone.0277670 (PMC9671377; doi:10.1371/journal.pone.0277670)

**Label-free Affinity Screening, Design and Synthesis of Inhibitors Targeting the *Mycobacterium tuberculosis* L-Alanine Dehydrogenase**

**Supporting information**

**S1 Method. Preparation of bone marrow-derived macrophages by *Mycobacterium tuberculosis*** **H37Rv** **infection and intracellular colony-forming unit assay.**

C57BL/6 mice (6 to 8-week-old) were purchased from Samtako Bio, Korea, and were maintained on a 12 h light/dark cycle under specific pathogen-free conditions. All mice were bred and housed for experiments in accordance with the guidelines of Chungnam National University School of Medicine. For the preparation of bone marrow-derived macrophages, mice were euthanized by CO_2_ asphyxiation. Mice experimental protocols were approved by the Institutional Animal Care and Use Committee of Chungnam National University (CNUH-A0011-1).

Bone marrow from the wild-type mice were harvested and cultured in Dulbecco’s modified Eagle’s medium (DMEM; Lonza; 12-604F) containing 10% fetal bovine serum (FBS; Gibco; 16000-044) for 3–5 days in the presence of 25 ng/ml of macrophage colony-stimulating factor (R&D Systems; 416-ML). Fully differentiated bone marrow-derived macrophage (BMDM) cells were infected with *Mycobacterium tuberculosis* (MOI = 1) for 4 h. Extracellular bacteria were washed with PBS and further cultured in the positive control 0.5 µg/ml isoniazid and the presence or absence of N6-methyl adenosine (500 μM) for 3 days.

For analysis of intracellular bacterial viability, the cells were lysed in distilled water to release intracellular bacteria. The serially diluted homogenates of the infected cells were plated in 7H10 agar plates, and colonies were counted after 21 days of incubation.

**S2 Method. Preparation of *Mycobacterium tuberculosis*** **H37Rv** **cells and minimum inhibitory concentration assay.**

H37Rv cell colonies were picked from Löwenstein–Jensen (LJ) medium and collected in u-shape bottom tube. The colonies were dispersed by vortexing with 2 mm-dia glass beads. After 15 min stabilization, the stock solution with McFarland 2.0~3.0 was prepared in 7H9 medium with 10% of OADC (BD science).

The 166 µl of cell stock solution was mixed with 500 µl of 0.5% liquid agarose at 37°C. Ten µl of the mixture was loaded into the inlet well to form microfluidic agarose channels surrounding the liquid medium loading well. After the cells were immobilized by gel solidification at 25°C, the positive control 0.5 µg/ml isoniazid and N6-methyl adenosine at 0 (negative control), 0.625, 1.25, 2.5, 5, 10, 20, 40, 80, 160 µg/ml concentrations in 7H9 medium with 10% OADC were added into the liquid medium loading wells to make it diffused towards the microfluidic agarose channels. The 96-well format MAC chip loaded with H37Rv and N6-methyl adenosine was incubated at 37°C and imaged same boundary area between the liquid medium loading well and microfluidic agarose channel by 40x lens of inverted microscopy with time-lapse of 1, 3, 5, 7 and 9 days. The collected images were analyzed to measure the minimum inhibitory concentration of N6-methyl adenosine based on the growth of H37Rv cells.

**S3 Method. Synthesis of adenosine derivative compounds, and analysis of their identity and purity by melting point, specific rotation, 1H NMR, 13C NMR, IR and high-resolution mass spectral (HRMS) data.**

Adenosine derivatives

For N6-alkyl adenosine (e.g. N6-methyl and N6-ethyl adenosine), a representative procedure is as following: A mixture of 6-chloro-9-β-D-ribofuranosyl-9H-purine (0.250 g, 0.87 mmol) and 33 wt% of methylamine in water (12 mL) was heated to 70-80^°^C in a closed reaction vessel overnight. The mixture was adsorbed onto silica gel and concentrated to dryness. The products were purified on silica gel with 10% MeOH/CH_2_Cl_2_ to provide 0.215 g (87%) of the title product as a white solid.

N6-methyl adenosine (> 95%): white solid; mp: 215.6-218.1℃; ${[\alpha]}_{D}^{25}$ = –9.8 (0.01 M in MeOH); ^1^H NMR (600 MHz , DMSO-*d*_6_): δ_H_ 8.33 (1H, s, H-8), 8.22 (1H, br. s, H-2), 7.81 (1H, br. s, NH-6), 5.87 (1H, d, J = 6.4 Hz, H-1′), 5.43 (1H, overlapped, OH-2′), 5.42 (1H, dd, J = 4.4, 7.2 Hz, OH-5′), 5.19 (1H, br. s, OH-3′), 4.60 (1H, br. dd, J = 6.4, 7.2 Hz, H-2′), 4.13 (1H, br.s, H-3′), 3.95 (1H, ddd, J = 2.8, 2.8, 3.6 Hz, H-4′), 3.66 (1H, ddd, J = 12.0, 3.2, 4.4 Hz, H-5′a), 3.53 (1H, ddd, J = 12.0, 2.8, 7.2 Hz, H-5′b), 2.95 (3H, br. s, H-1ʺ); ^13^C NMR (150 MHz, DMSO-d_6_): δ_C_ 155.06 (C-6), 152.43 (C-2), 147.80 (C-4), 139.65 (C-8), 119.90 (C-5), 87.91 (C-1′), 85.90 (C-4′), 73.47 (C-2′), 70.66 (C-3′), 61.67 (C-5′), 26.96 (N6-CH_3_); IR (KBr): 3483 (OH), 3139 (NH), 1630, 1588 (aromatic) cm^-1^; HRMS (m/z): [M+H]^+^ calcd. for C_11_H_16_N_5_O_4_, 282.1197; found, 282.1191.

N6-ethyl adenosine (99%): white solid; mp: 197.1-198.4℃; ${[\alpha]}_{D}^{25}$ = –3.6 (0.01 M in MeOH); ^1^H NMR (600 MHz, DMSO-d_6_): δ_H_ 8.34 (1H, s, H-8), 8.21 (1H, br. s, H-2), 7.87 (1H, br. s, NH-6), 5.88 (1H, d, J = 6.0 Hz, H-1′), 5.46 (1H, overlapped, OH-2′), 5.44 (1H, dd, J = 4.4, 7.6 Hz, OH-5′), 5.21 (1H, br. s, OH-3′), 4.61 (1H, br. dd, J = 6.0, 7.2 Hz, H-2′), 4.15 (1H, br.s, H-3′), 3.97 (1H, ddd, J = 2.8, 2.8, 3.2 Hz, H-4′), 3.68 (1H, ddd, J = 12.4, 3.2, 4.4 Hz, H-5′a), 3.55 (1H, ddd, J = 12.4, 2.9, 7.6 Hz, H-5′b), 3.51 (2H, overlapped, H-1ʺ) 1.18 (3H, t, J = 5.4 Hz, H-2ʺ); ^13^C NMR (150 MHz, DMSO-d_6_): δ_C_ 154.49 (C-6), 152.38 (C-2), 148.22 (C-4), 139.65 (C-8), 119.78 (C-5), 87.93 (C-1′), 85.90 (C-4′), 73.45 (C-2′), 70.66 (C-3′), 61.68 (C-5′), 34.53 (N6-CH_2_), 14.77 (N6-CH_3_); IR (KBr): 3324 (OH), 3149 (NH), 1622, 1589 (aromatic) cm^-1^; HRMS (m/z): [M+H]^+^ calcd. for C_12_H_18_N_5_O_4_, 296.1353; found, 296.1349.

For N6-propyl and N6-isobutyl adenosine, a representative procedure is as following:

A mixture of 6-chloro-9-β-D-ribofuranosyl-9H-purine (0.250 g, 0.87 mmol), isobutylamine (0.195 g, 2.64 mmol) and CaCO_3_ (0.175 g, 1.75 mmol) in EtOH (12.5 mL) was heated to reflux overnight. The reaction mixture was filtered while hot and hot EtOH was used to wash the solids. After cooled to room temperature, the precipitate was collected by filtration, washed with cold EtOH and dried under reduced pressure to give the title compound (0.195 g, 69%) as a white solid. The filtrate was adsorbed on silica gel and was concentrated to dryness. This mixture was purified on silica gel with 10% MeOH/CH_2_Cl_2_ to provide 0.091 g more of the compound for a quantitative yield of product.

N6-propyl adenosine (> 95%): white solid; mp: 162.0-165.4℃; ${[\alpha]}_{D}^{25}$ : –8.8 (0.01 M in MeOH); ^1^H NMR (600 MHz, DMSO-d_6_): δ_H_ 8.34 (1H, s, H-8), 8.21 (1H, br. s, H-2), 7.91 (1H, br. s, NH-6), 5.88 (1H, d, J = 6.4 Hz, H-1′), 5.47 (1H, overlapped, OH-2′), 5.44 (1H, dd, J = 4.8, 7.2 Hz, OH-5′), 5.22 (1H, br. s, OH-3′), 4.62 (1H, br. dd, J = 6.4, 5.2 Hz, H-2′), 4.16 (1H, br.s, H-3′), 3.96 (1H, ddd, J = 3.2, 3.2, 3.2 Hz, H-4′), 3.68 (1H, ddd, J = 12.0, 3.2, 3.2 Hz, H-5′a), 3.55 (1H, ddd, J = 12.0, 3.2, 7.2 Hz, H-5′b), 3.44 (2H, br. s, H-1ʺ) 1.61 (2H, tq, J = 7.2, 7.2 Hz, H-2ʺ), 0.90 (3H, t, J = 7.2 Hz, H-3ʺ); ^13^C NMR (150 MHz, DMSO-d_6_): δ_C_ 154.73 (C-6), 152.38 (C-2), 148.22 (C-4), 139.64 (C-8), 119.73 (C-5), 87.95 (C-1′), 85.91 (C-4′), 73.44 (C-2′), 70.67 (C-3′), 61.69 (C-5′), 41.47 (N6-CH_2_), 22.28 (N6-CH_2_), 11.32 (N6-CH_3_); IR (KBr): 3333 (OH), 3150 (NH), 1626, 1588 (aromatic) cm^-1^; HRMS (m/z): [M+H]^+^ calcd. for C_13_H_20_N_5_O_4_, 310.1510; found, 310.1502.

N6-isobutyl adenosine (99%): white solid; mp: 160.3-162.9℃; ${[\alpha]}_{D}^{25}$ = –5.0 (0.01 M in MeOH); ^1^H NMR (600 MHz, DMSO-d_6_): δ_H_ 8.35 (1H, s, H-8), 8.20 (1H, br. s, H-2), 7.94 (1H, br. s, NH-6), 5.88 (1H, d, J = 6.0 Hz, H-1′), 5.46 (1H, overlapped, OH-2′), 5.44 (1H, dd, J = 4.4, 7.6 Hz, OH-5′), 5.21 (1H, br. s, OH-3′), 4.62 (1H, t-like, H-2′), 4.15 (1H, m, H-3′, 3.97 (1H, ddd, J = 3.6, 3.6, 3.2 Hz, H-4′), 3.68 (1H, ddd, J = 12.4, 3.6, 4.4 Hz, H-5′a), 3.55 (1H, ddd, J = 12.4, 3.6, 7.6 Hz, H-5′b), 3.30 (2H, overlapped, H-1ʺ) 1.97 (1H, m, H-2ʺ), 0.89 (6H, d, J = 6.8, Hz, H-3ʺ, H-4ʺ); ^13^C NMR (150 MHz, DMSO-d_6_): δ_C_ 154.84 (C-6), 152.33 (C-2), 148.21 (C-4), 139.65 (C-8), 119.69 (C-5), 87.96(C-1′), 85.92 (C-4′), 73.42 (C-2′), 70.68 (C-3′), 61.63 (C-5′), 47.15 (N6-CH_2_), 27.79 (N6-CH), 20.11 (x 2, N6-CH_3_); IR (KBr): 3550 (OH), 3339, 3151 (NH), 1625, 1590 (aromatic) cm^-1^; HRMS (m/z): [M+H]^+^ calcd. for C_14_H_22_N_5_O_4_, 324.1666; found, 324.1659.

A representative procedure for N6-acyl adenosine (e.g. N6-acetyl and N6-isobutyroyl adenosine) is as following: To a suspension of adenosine (2.0 g, 7.48 mmol, coevaporated three times with pyridine and then dried under high vacuum) in pyridine (15.0 mL) was added dropwise chlorotrimethylsilane (8.1 g, 74.54 mmol) via a syringe pump at 4^o^C. The mixture was allowed to warm to room temperature and stirred overnight. Acetonitrile (22 mL) was added and the mixture was cooled to 4^o^C, acetyl chloride (0.883 g, 11.25 mmol) was then added. The mixture was stirred at this temperature for 1 h and diluted with dichloro-methane (44 mL) and added to water (30 mL, 4^o^C) with stirring. The organic phase was washed with water (40 mL, 4^o^C), dried over NaSO_4_, evaporated and co-evaporated with toluene until all pyridine was removed. The residue was dissolved in MeOH (15 mL) and treated with AcOH (4.5 mL) and stored at -20^o^C for 2 days. The solvent and excess reagents were removed by rotary evaporation and the residue was adsorbed onto silica gel. The product was obtained by chromatography on silica gel with 20% MeOH/CH_2_Cl_2_ to a white foaming solid (1.50 g, 65%).

N6-acetyl adenosine (> 95%): white solid; mp: 167.7-169.3℃; ${[\alpha]}_{D}^{25}$ = –2.3 (0.01 M in MeOH); ^1^H NMR (600 MHz, DMSO-d_6_): δ_H_ 8.66 (1H, s, H-8), 8.71 (1H, s, H-2), 6.01 (1H, d, J = 6.0 Hz, H-1′), 5.57 (1H, br. s, OH-2′), 5.28 (1H, br. s, OH-5′), 5.15 (1H, br. dd, J = 5.4, 5.4 Hz, OH-3′), 4.63 (1H, t-like, H-2′), 4.19 (1H, br. s, H-3′), 3.98 (1H, ddd, J = 4.0, 4.0 3.6 Hz, H-4′), 3.70 (1H, ddd, J = 12.0, 4.0, 4.0 Hz, H-5′a), 3.57 (1H, overlapped, H-5b′), 2.26 (3H, s, H-1ʺ); ^13^C NMR (150 MHz, DMSO-d_6_): δ_C_ 168.86 (N6-acetyl ester), 151.74 (C-6), 151.65 (C-2), 149.62 (C-4), 142.81 (C-8), 123.73 (C-5), 87.60 (C-1’), 85.72 (C-4’), 73.65 (C-2’), 70.35 (C-3’), 61.32 (C-5’), 24.31 (N6-acetyl-CH_3_); IR (KBr): 3323 (OH), 3107 (NH), 1651, 1581 (aromatic) 3319 (OH), 3135 (NH), 1685 (amide), 1612, 1587 (aromatic); HRMS (m/z): [M+H]^+^ calcd. for C_12_H_16_N_5_O_5_, 310.1146; found, 310.1141.

N6-isobutyroyl adenosine (> 95%): white solid; mp: 109.4-113.2℃; ${[\alpha]}_{D}^{25}$ = –1.0 (0.01 M in MeOH); ^1^H NMR (600 MHz, DMSO-d_6_): δ_H_ 8.69 (1H, s, H-8), 8.55 (1H, s, H-2), 6.01 (1H, d, J = 6.0 Hz, H-1′), 5.58 (1H, br. s, OH-2′), 5.29 (1H, br. s, OH-5′), 5.15 (1H, br. dd, J = 5.4, 5.4 Hz, OH-3′), 4.63 (1H, br. dd, J = 6.0, 5.2 Hz, H-2′), 4.19 (1H, br. s, H-3′), 3.98 (1H, ddd, J = 3.6, 3.6, 4.0 Hz, H-4′), 3.69 (1H, ddd, J = 12.0, 3.6, 4.0 Hz, H-5′a), 3.57 (1H, overlapped, H-5b′), 2.97 (1H, septet, J = 6.8 Hz, H-1ʺ), 1.14 (6H, d, J = 6.8 Hz, H-2ʺ, H-3ʺ); ^13^C NMR (150 MHz, DMSO-d_6_): δ_C_ 175.37 (N6-isobutyroyl ester), 151.82 (C-6), 151.64 (C-2), 149.89 (C-4), 142.77 (C-8), 124.19 (C-5), 87.62 (C-1’), 85.70(C-4’), 73.66 (C-2’), 70.33 (C-3’), 61.32 (C-5’), 34.36 (N6-isobutyroyl-CH), 19.26 (N6-isobutyroyl-CH_3_), 19.24 (N6-isobutyroyl-CH_3_); IR (KBr): 3263 (OH), 1716 (amide), 1609, 1584 (aromatic) 2360, 1706, 1630 cm^-1^; HRMS (m/z): [M+H]^+^ calcd. for C_14_H_20_N_5_O_5_, 338.1459; found, 338.1455.

**S4 Method. Docking simulation analysis by CCDC and DSX.**

**(i) Score potential analysis of N6-isobutyl adenosine for AlaDH structure by DSX.** From the pdb file of AlaDH structure solved with N6-isobutyl ado (PDB code, 6O7F), the pdb files of each N6-isobutyl ado structure and AlaDH structure were derived, and the pdb file of N6-isobutyl ado was converted to mol2 format file after adding hydrogens by using CCDC Hermes. The pdb file of N6-methyl ado derived from the pdb file of AlaDH structure solved with N6-methyl ado (PDB code, 4LMP) was also converted to mol2 format file after adding hydrogens by using CCDC Hermes. Then, the DSX online (v0.88) program was opened, and in ‘New DSX-ONLINE Job’ menu, the input structure files was provided by browsing the pdb file of AlaDH structure as a protein file, the mol2 file of N6-isobutyl ado as a ligand(s) file, and the mol2 file of N6-methyl ado as a reference ligand file. For the source of pair potentials, CSD was chosen, and option 1 of cofactors was selected, waters and metals were treated as part of the protein for interaction mode. By submitting data after choosing ‘SCORE Gold-WATER’ and ‘SCORE TORSIONS’ as well as ‘VISUALIZE’ in ‘Further Options’ menu, the job was initiated. Once the submitted job was finished, the resulted data files of DSX protein file, DSX ligand file, DSX reference file, DSX results file and DSX visualization file were downloaded. To visualize the (good and bad) score potentials of protein and ligand and (good and bad) distances between protein and ligand in Pymol, the downloaded py file was opened after selecting ‘All Files in Files’ of type together with the protein pdb file and the ligand mol2 files.

**(ii) Docking and score potential analysis of N6-isobutyl NAD for human dehydrogenases.** Thirteen pdb files of human DH structures solved with NAD (PDB codes, 1F0Y, 1MP0, 1O01, 1PL8, 1X0X, 1ZMC, 2GDZ, 2I9P, 4OKN, 4WLV, 5C7O, 5FHZ, 5JS6) were downloaded from the PDB. From each NAD and human DH complex structure pdb file, the individual pdb files of NAD structure and human DH structure were created by file editing, and the pdb file of NAD was converted to mol2 file by using the CCDC Hermes program.

In the CCDC Hermes, by using each set of NAD mol2 file and human DH pdb file, the NAD structure mol2 file was uploaded, and the isobutyl group was added at N6 of NAD by ‘Edit Structure’ function and hydrogens were added in Edit menu, and N6-isobutyl NAD structure was saved in mol2 format. After opening human DH protein pdb file, N6-isobutyl NAD mol2 file and NAD mol2 file in the CCDC Hermes to show all three structures in the Hermes screen, the GOLD program was opened by choosing ‘Setup and Run a Docking’. In ‘Proteins’ menu under ‘Global Options’, the target human DH was selected to open the new tab which contains ‘Add Hydrogens’ button to add hydrogens to the protein structure and to see the number of added hydrogens. Then, in ‘Define Binding Site’ menu under ‘Global Options’, ‘One or more ligands’ was chosen and the NAD file was selected. In ‘Select Ligands’ menu, add N6-isobutyl ado mol2 file. In ‘Ligand Flexibility’, ‘Flip all planar R-NR1R2’ was chosen by clicking ‘Flip protonated carboxylic acids’ to set all for flip, and the default for ‘Use Torsion Angle Distributions’ and ‘Postprocess Rotatable Bonds’ were selected. The ‘Fix Ligand Rotational Bonds’ was clicked to fix specific bonds by ‘Specify Bonds’ button; after setting the Hermes visualizer screen to show only the structure of N6-isobutyl NAD, all bonds of NAD part were fixed in the pop-up window of ‘Select Ligand Bonds To Fix’ by selection of the bond with left mouse button and fix it with right mouse button menu for ‘Fix Bond’. In ‘Fitness & Search Option’, the ‘Docking’ was selected by choosing ‘Chemscore’ for Scoring Function with the default for Parameter file, and by allowing early termination if the top 3 solutions are within 1.5 Å as ‘Early Termination Options’, and by using the internal ligand energy offset. The automatic was chosen in ‘GA settings’ menu. In ‘Output Options’, output file format was set as mol2, and output directory and save files were assigned by clicking all file options in ‘File Format Options’, including all information in file in ‘Information in File’ and keeping all solutions in ‘Selecting Solutions’. For ‘Constrains’, ‘Never dock a ligand when a constraint is physically impossible’ was selected, and for ‘Atom typing’, Ligand was chosen. After setting of all these parameters, the docking process was started by ‘Run Gold’ in ‘The Background’ menu at the bottom. With the pop-up window of ‘Finish GOLD Configuration’, the output directory was checked to save GOLD configuration file and protein file with the correct names.

In the resulted .rnk file, the best scored N6-isobutyl NAD docking file was used to analyze score potential for each human DH protein structure by the DSX online program.


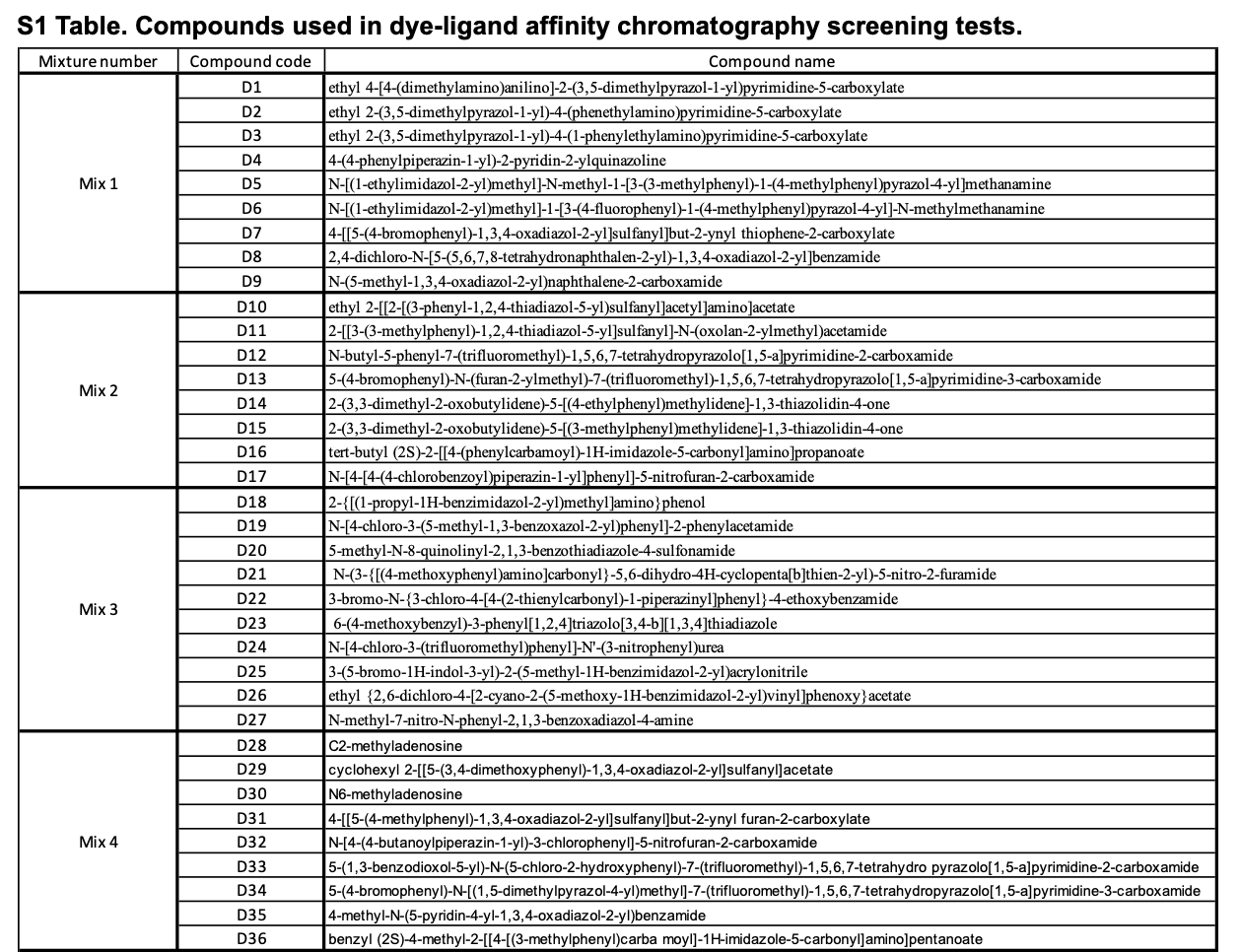

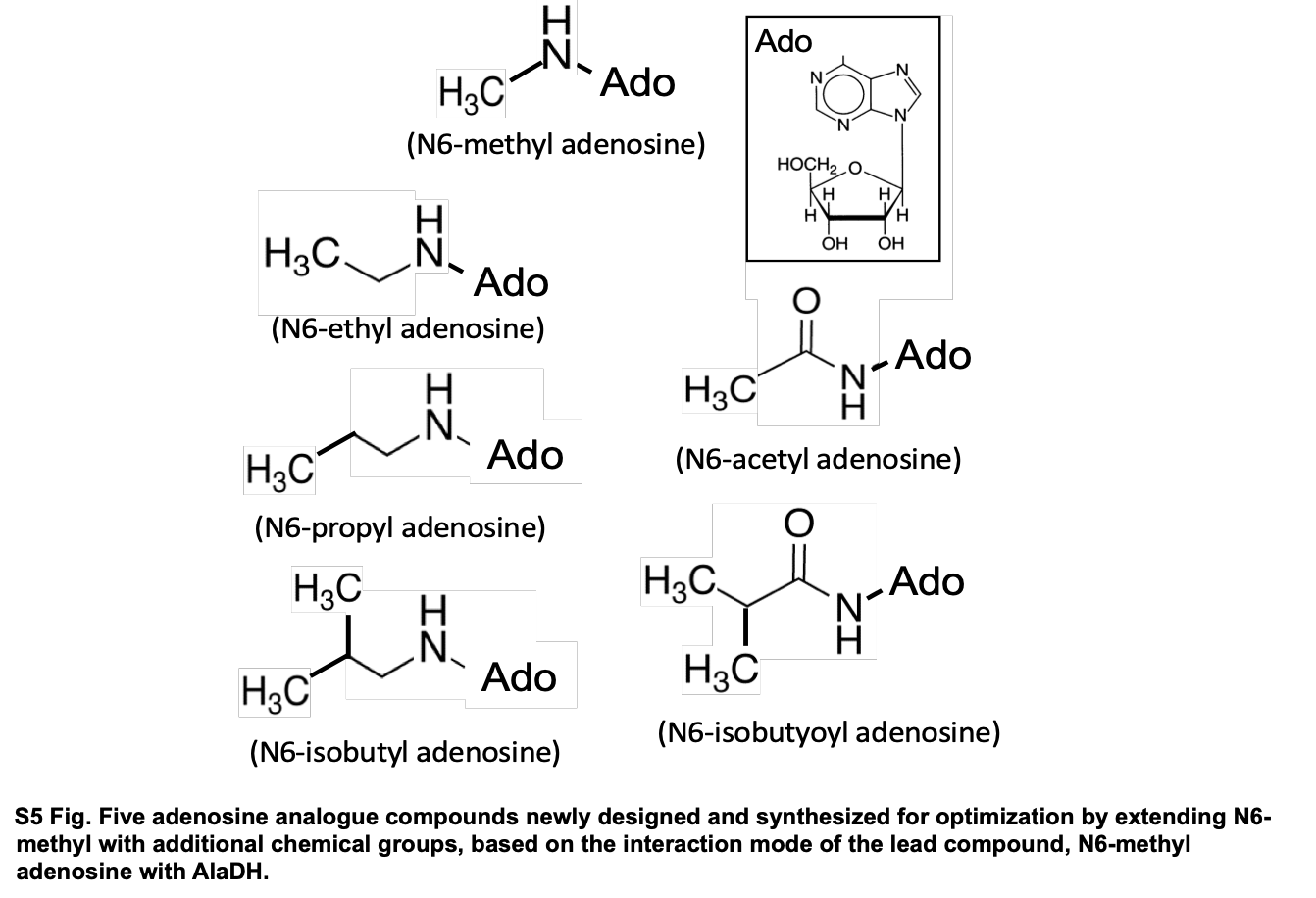

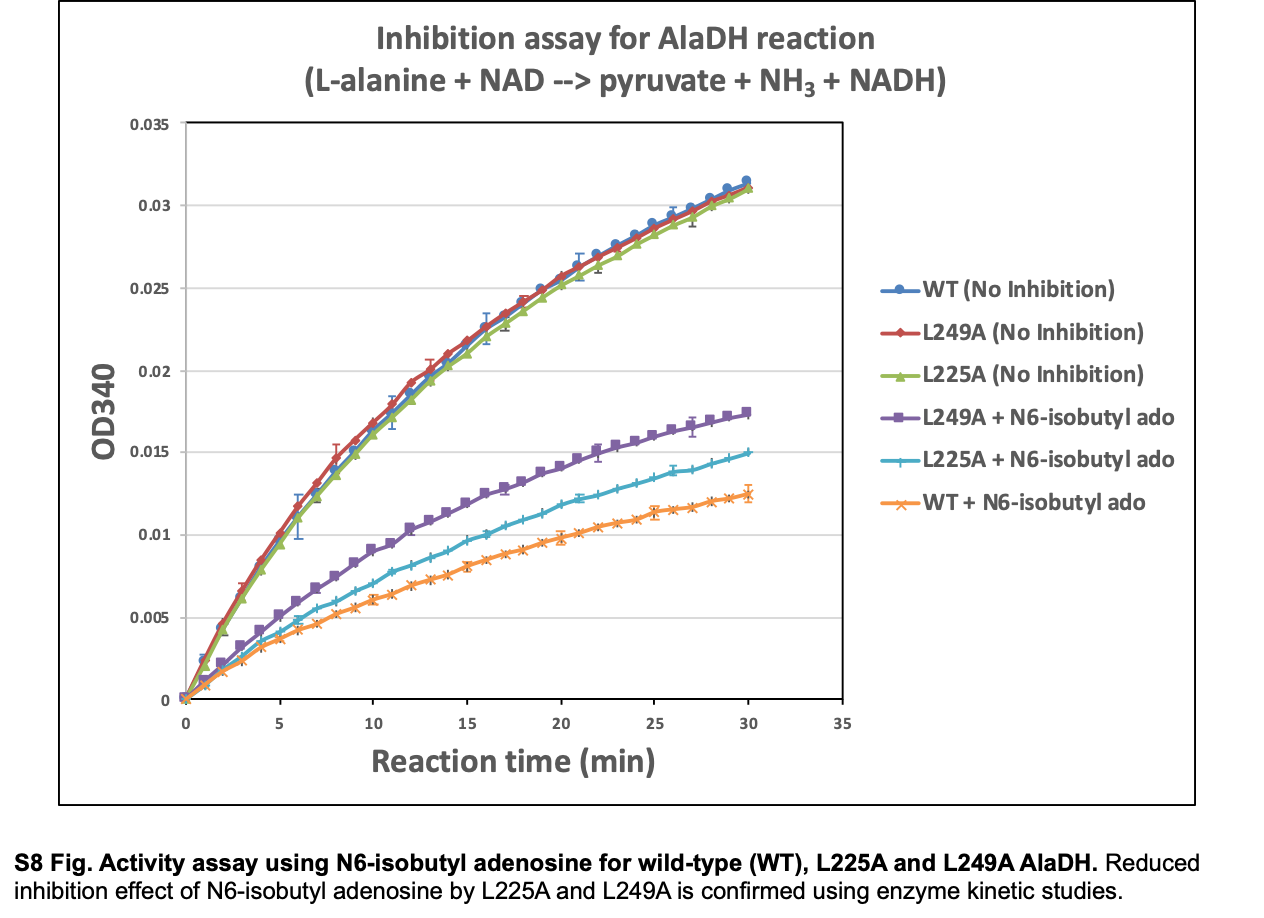


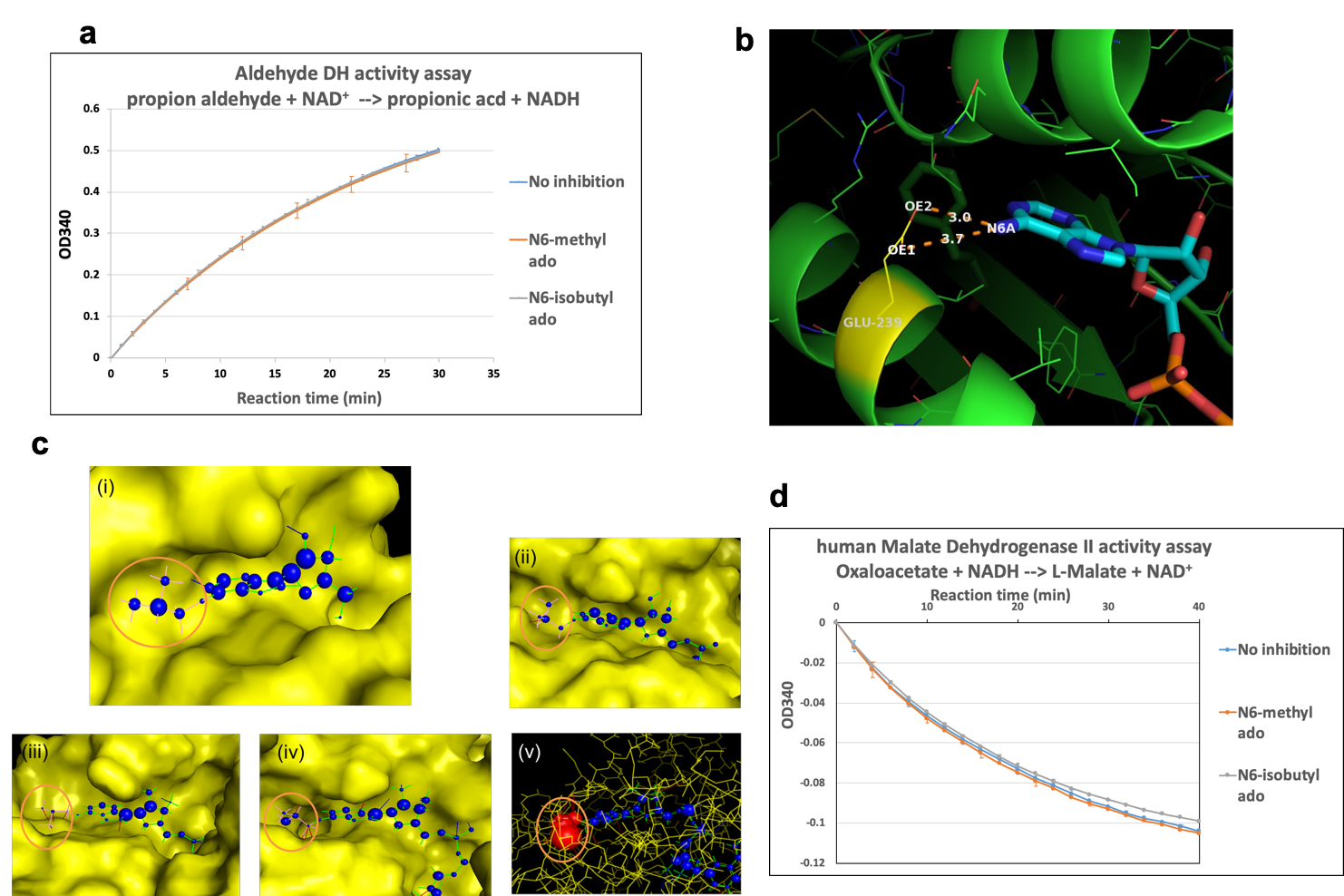


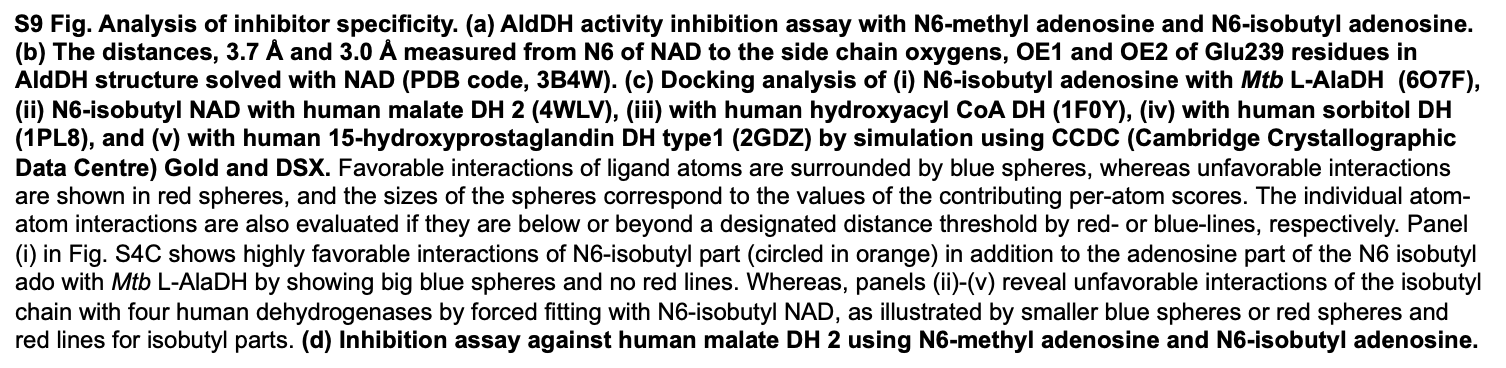

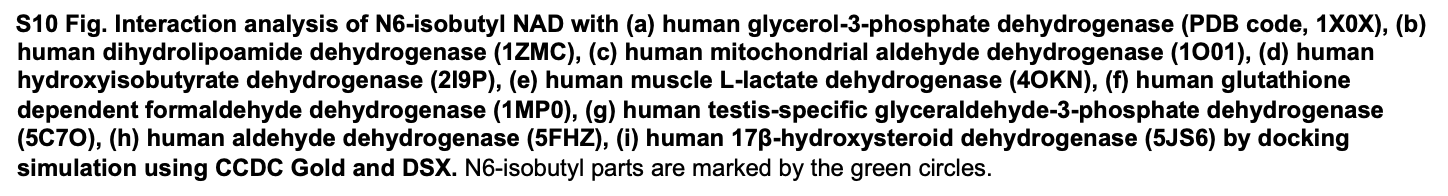


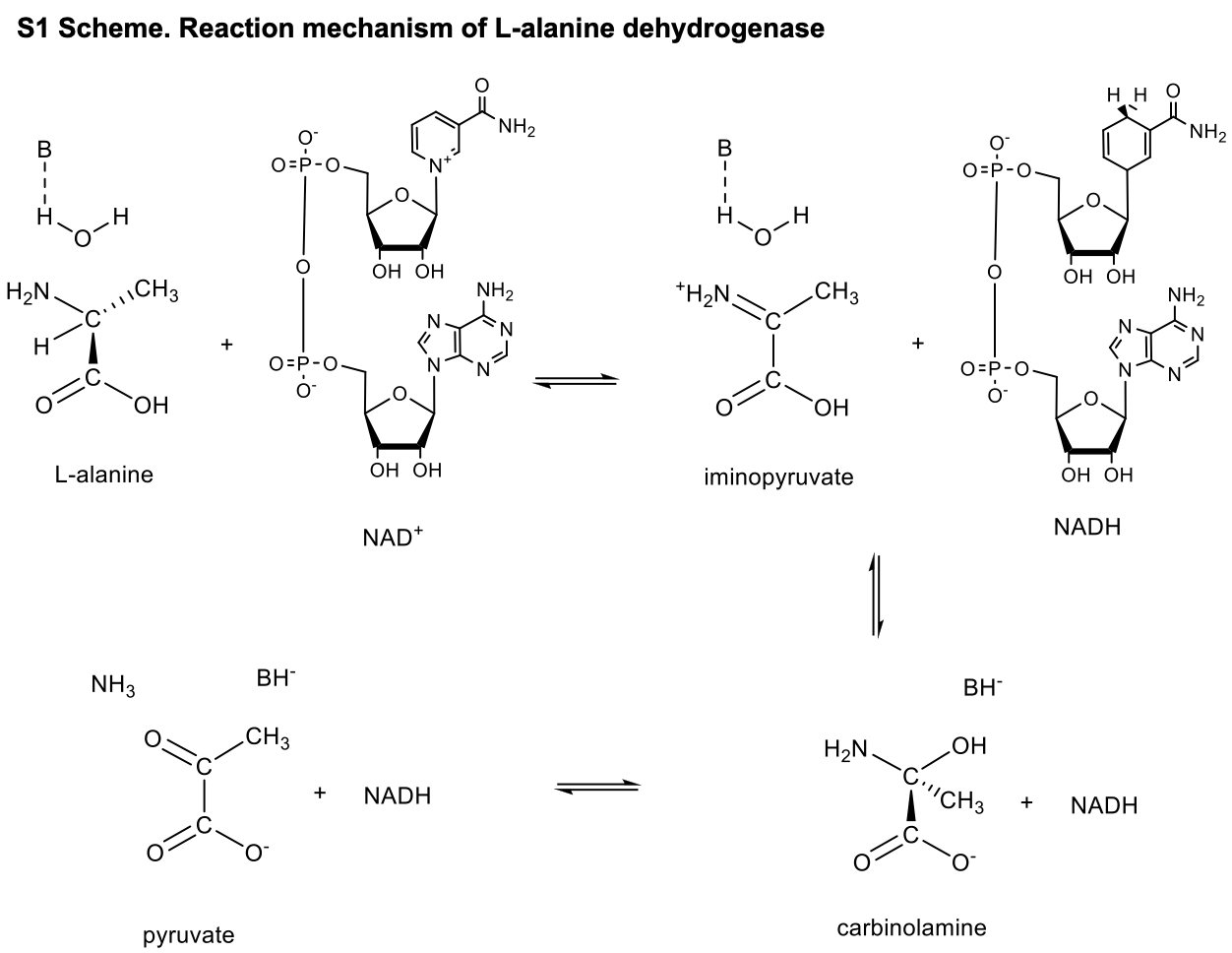


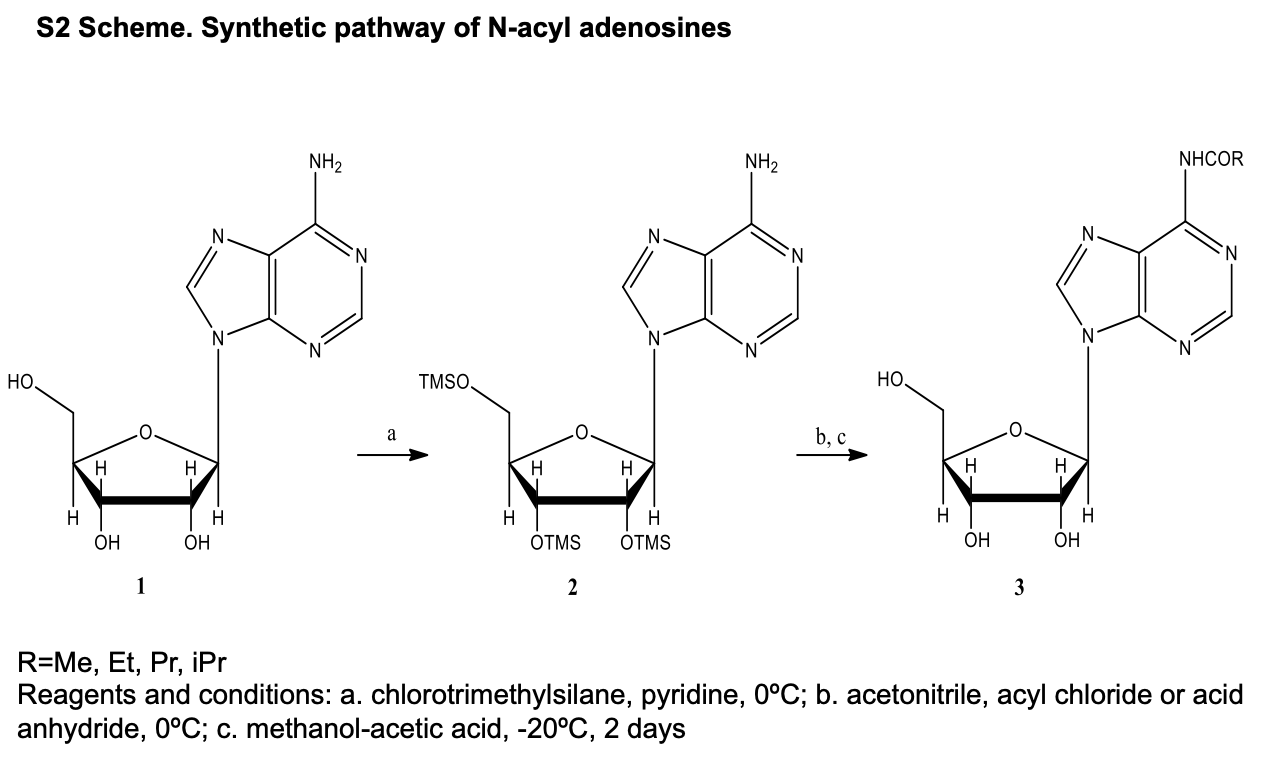


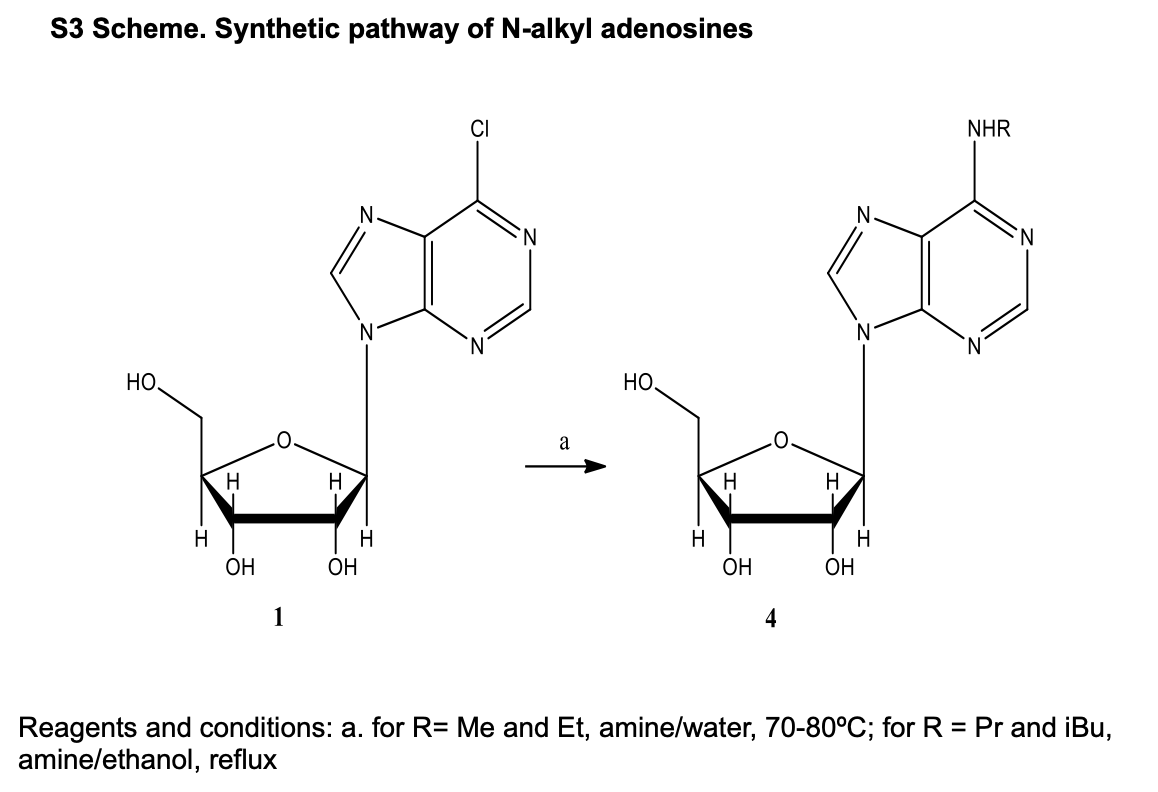

Supplement: S1 File — (DOCX) [file pone.0277670.s001.docx]
